# D-galactose-induced mitochondrial oxidative damage and apoptosis in the cochlear stria vascularis of mice

**DOI:** 10.1186/s12860-023-00480-7

**Published:** 2023-08-21

**Authors:** Zhe Peng, Chunli Zhao, Zijing Yang, Shusheng Gong, Zhengde Du

**Affiliations:** 1 Department of Otolaryngology Head and Neck Surgery, Beijing Friendship Hospital, Capital Medical University, No.95, Yong’an Road, Xicheng District, Beijing, 100050 China; 2 Clinical Center for Hearing Loss, Capital Medical University, Beijing, 100050 China

**Keywords:** Mitochondrial oxidative damage, Apoptosis, Stria vascularis, D-galactose, Mouse

## Abstract

**Background:**

Age-related hearing loss, known as presbycusis, is the result of auditory system degeneration. Numerous studies have suggested that reactive oxygen species (ROS) and mitochondrial oxidative damage play important roles in the occurrence and progression of aging. The D-galactose (D-gal)-induced aging model is well known and widely utilized in aging research. Our previous studies demonstrate that administration of D-gal causes mitochondrial oxidative damage and causes subsequent dysfunction in the cochlear ribbon synapses, which in turn leads to hearing changes and early stage presbycusis. Stria vascularis (SV) cells are vital for hearing function. However, it is unclear to what extent D-gal induces oxidative damage and apoptosis in the cochlear SV of mice. In addition, the source of the causative ROS in the cochlear SV has not been fully investigated.

**Methods:**

In this study, we investigated ROS generation in the cochlear SV of mice treated with D-gal. Hearing function was measured using the auditory brainstem response (ABR). Immunofluorescence was used to examine apoptosis and oxidative damage. Transmission electron microscopy was also used to investigate the mitochondrial ultrastructure. DNA fragmentation was determined using the terminal deoxynucleotidyl transferase-mediated dUTP-biotin nick end-labeling (TUNEL) assay. Mitochondrial membrane potential (MMP) and ATP were also measured.

**Results:**

We found that D-gal-treated mice exhibited a significant shift in the mean amplitude and latency of the ABR; a remarkable increase in the levels of NADPH oxidase (NOX-2), Uncoupling protein 2 (UCP2) and cleaved caspase-3 (c-Cas3) was observed, as well as an increase in the number of TUNEL-positive cells were observed in the SV of mice. Both the expression of the DNA oxidative damage biomarker 8-hydroxy-2-deoxyguanosine (8-OHdG) and a commonly occurring mitochondrial DNA deletion were markedly elevated in the SV of mice that had been treated with D-gal to induce aging. Conversely, the ATP level and MMP were significantly reduced in D-gal-induced aging mice. We also found alterations in the mitochondrial ultrastructure in the SV of aging mice, which include swollen and distorted mitochondrial shape, shortened and thickened microvilli, and the accumulation of lysosomes in the SV.

**Conclusion:**

Our findings suggest that the impairment of cochlear SV during presbycusis may be caused by mitochondrial oxidative damage and subsequent apoptosis.

## Introduction

Age-related hearing loss (ARHL), also known as presbycusis, is the progressive loss of hearing associated with aging and is the most common sensory disorder in the elderly population [1–4]. There are four types of ARHL involving the cochlea: sensory, neural, strial/metabolic, and cochlear conductive, according to Schuknecht’s pioneering work. Strial or metabolic presbycusis is the loss of stria vascularis (SV) cells that impairs blood supply to the inner ear, which causes hearing loss that manifests across all frequencies [5]. The hypothesis that vascular degeneration can play a role in presbycusis is widely held concept [1, 6, 7]; however, the underlying biological mechanism remains unclear. Therefore, numerous animal models have been developed to promote the study of molecular mechanisms of ARHL. Animal models of D-galactose (D-gal) were widely used for this purpose [8–13]. D-gal is a common reducing sugar in vivo. At normal levels, galactose-1-phosphate uridyltransferase and galactokinase convert it to glucose. At high concentrations, however, it can be oxidized to aldehydes and H_2_O_2_ in the presence of galactose oxidase, causing oxidative stress in vivo [14].

In most mammalian cells, reactive oxygen species, including hydroxyl radicals H_2_O_2_, are produced mainly by mitochondria [15, 16]. A growing body of research indicates that reactive oxygen species (ROS) and oxidative stress play an important role in the pathogenesis of various cochlear abnormalities, especially sensorineural hearing loss, which can be induced by ototoxicity, senility, and noise exposure [17–19].

Some studies have demonstrated that D-gal-treated animal models exhibit increased oxidative stress levels and an accumulation of mitochondrial DNA (mtDNA) common deletion (CD) in both the peripheral and central auditory systems [20–22]. A previous study on D-gal-induced aging found that only a small number of marginal cells in the cochlear SV are lost due to the activation of mitochondria-dependent apoptosis [11]. The SV is a highly vascularized tissue that lines the cochlear lateral wall. The SV regulates cochlear fluid homeostasis and produces the endo-cochlear potential for sound transmission. Moreover, it acts as an important blood-labyrinth barrier, tightly regulating the passage of molecules from the blood into the cochlea. Therefore, a healthy SV is vital for hearing function [23]. However, few studies have systematically studied D-gal-induced oxidative damage and apoptosis in the mitochondria of the cochlear SV of mice. In addition, the source of the ROS that causes cochlear SV impairment has not been fully investigated.

Here, we investigated the expression of nitrous oxide (NOX2), uncoupling protein 2 (UCP2), and the DNA oxidative damage biomarker 8-hydroxy-2-deoxyguanosine (8-OHdG) in the SV, as well as changes in mitochondrial membrane potential (MMP), ATP, and mtDNA CD levels were investigated in mice with D-gal-induced aging. To determine the activity of caspase-3, the proteolytic cleavage product of caspase-3 (an N-terminated peptide substrate) was evaluated. DNA fragmentation in the nucleus was detected by terminal deoxynucleotidyl transferase-mediated dUTP-biotin nick end-labeling (TUNEL). The ultrastructural variation of target cells was observed by transmission electron microscopy (TEM).

In this study, we hypothesized that nitric oxide and mitochondria-dependent ROS generation, mtDNA oxidative damage, and apoptosis in cochlear SV may be among the primary causes of auditory system degeneration in D-gal induced aging.

## Materials and methods

### Animals

60 male C57BL/6J mice aged 5 weeks were obtained from Capital Medical University’s Experimental Animal Center. The mice were raised in an environment at a constant temperature (22–23 °C) and humidity (60 ± 5%), on a 12-h light/dark cycle, with free access to food and water. All mice were acclimated for a week before being divided into three groups (n = 20 per group): control, low-dose D-gal (D-gal-L) and high-dose D-gal (D-gal-H) groups. The mice in the control group received a subcutaneous injection of 0.9% saline (the D-gal vehicle) once daily for six weeks. The second and third groups of mice received a subcutaneous injection of D-gal (Sigma-Aldrich, St. Louis, MO, USA) once daily for 6 weeks at a dose of 500 and 1000 mg/kg body weight, respectively. In compliance with the National Institutes of Health’s Guidelines for the Care and Use of Laboratory Animals, every effort was made to minimize animal suffering and reduce the number of animals used. The Capital Medical University Committee on the Ethics of Animal Experiments approved the study’s protocol.

### Auditory function evaluation

Auditory brainstem response (ABR) was used to analyze the hearing function in all animals. The method of ABR testing has been described previously [24]. Briefly, it was conducted in an anechoic room using BioSigRZ software (Tucker-Davis Technologies, Alachua, FL, USA). A total of 18 mice (n = 6 per group) were anesthetized by intraperitoneal injection of a ketamine (100 mg/kg) and xylazine (10 mg/kg) mixture. Before testing, the external auditory canal and tympanic membrane (TM) were examined with an electric otoscope. Mice with acute otitis externa (AOE) or otitis media were excluded from the study.

The sound delivery tube of an inserted earphone was tightly fitted into the external auditory canal. Subcutaneous needle electrodes were used to record the ABR response. Three stainless steel recording electrodes were subcutaneously inserted posterior to the tested pinna (recording), vertex (reference), and contralateral pinna (ground). A TDT System 3 (Tucker-Davis Technologies) was used to measure ABR responses to a tone burst stimulus at 8 kHz, 16 and 32 kHz, starting at a 90-dB sound pressure level (SPL) and decreasing in 5-dB steps. The ABR threshold for each frequency was determined, which is the lowest SPL that will reliably produce an ABR recording with one or more distinct waves that can be easily distinguished by visual inspection of the waveforms. To confirm the uniformity of the waveforms, the procedure was repeated at low SPLs close to the threshold.

### Immunohistochemistry of cochlear frozen sections

Mice (18 per group, n = 6) were anesthetized with ketamine and xylazine before being euthanized via cervical dislocation. Cochleae were fixed with 4% paraformaldehyde (PFA) overnight at 4 °C, then washed three times with PBS before being decalcified in 10% EDTA for 48 h at 4 °C. Samples were dehydrated in a sucrose gradient of 20 and 30% for 1 h each. Samples were embedded in optimal cutting temperature compound before being sliced at 20 °C with a thickness of 10 μm using a Leica Cryastat (Wetzlar, Germany) and mounted on Superfrost Plus microscopic slides (KeyGEN Biotech, Nanjing, China). One side of the cochleae were used for immunohistochemistry, and the other side for TUNEL staining. The expression of NOX2, UCP2, 8-OHdG, cleaved caspase 3 (c-Cas3) were evaluated using immunohistochemistry. Cochlear sections were washed and then incubated with 0.3% Triton X-100 solution (Sigma-Aldrich) for 30 min at room temperature. They were washed again and blocked with 10% goat serum for 1 h at room temperature and incubated overnight at 4 °C with primary antibodies: monoclonal mouse anti-8-OHdG (diluted 1:200; Abcam, USA, Cat.No#ab62623), anti-NOX2 (diluted 1:200; Invitrogen, Waltham, MA, USA, Cat.No#PA5-76034,), anti-UCP2 (diluted 1:200; Invitrogen,Cat.No#PA5-103176) and anti-C-cas3 (diluted 1:200; CST, Danvers, MA, USA, Cat.No#Asp175). The slides were then washed and incubated in the dark for 2 h with Alexa Fluor 568-, Alexa Fluor 488-, or Alexa Fluor 594-conjugated secondary antibodies at a dilution of 1:300. The sections were washed and mounted using mounting media containing 4′,6-diamidino-2-phenylindole (DAPI; ZSGB-BIO, Beijing, China). Following a wash with PBS, the sections were examined using a laser scanning confocal microscope (Leica TCS SP8). The expression levels of 8-OHdG, NOX2, UCP2 and c-Cas3 were analyzed using the Image-Pro Plus 6.0 software (Media Cybernetics, Rockville, MD, USA). Sections were treated the same way in the negative control, but the primary antibody incubation step was skipped.

### DNA isolation and mitochondrial DNA (mtDNA) 3860-bp deletion assay

The method of DNA isolation and mtDNA deletion has been described previously [25, 26]. The accumulation of mtDNA was evaluated in 12 mice (n = 4 per group) using real-time PCR assay. DNA extraction by using DNA isolation kit (Tiangen Biotech Co., Beijing, China). GeneQuant pro DNA/RNA Calculator (Amersham Pharmacia Biotech, Staffanstorp, Sweden) was used to measure DNA concentration. 12 S rRNA gene was used as control (GenBank: NC_006914). The primers used to amplify the mtDNA 3860-bp deletion and 12 S rRNA were previously described [25]. PCR amplification was performed on a StepOne Real-Time PCR System (Applied Biosystems, Waltham, MA, USA). The cycling conditions were as follows: 95 °C for 10 min, 40 cycles at 95 °C for 15 s and 60 °C for 1 min. The cycle number at which a significant increase in normalized fluorescence was first detected was designated as the threshold cycle number (Ct). The ratio of the mtDNA 3860-bp deletion to total mtDNA was calculated as ΔCt = (Ct3860-bp deletion − Ct12S rRNA), and the relative expression was calculated as 2 − ΔΔCt. The PCR products of the mtDNA 3860-bp deletion were cloned and verified with ABI Prism 377XL sequencer (Applied Biosystems).

### Transmission electron microscopy

The ultrastructure of the mitochondria in the cochlea SV was observed using TEM. Twelve mice (n = 4 per group) were anesthetized with ketamine and xylazine before being euthanized via cervical dislocation. Then cochleae from each mouse were removed, treated with 2.5% glutaraldehyde and fixed overnight at 4 °C. The following day, the cochleae were washed with PBS and placed in 10% EDTA for decalcification for 72 h. The SV was carefully dissected and harvested from the lateral wall of the cochlea. After fixation in 1% osmium tetroxide (Maijin Biotechnology, Nanjing, China) for 2 h, the SV was dehydrated using graded ethanol or acetone, followed by a 2-h immersion in acetone/Epon 812 mixture (1:1), and a 10-h immersion in Epon 812 at 80 °C. Ultrathin Sect. (50 nm) were taken serially on copper grids and were stained with uranyl acetate and lead citrate., A FEI TecnaiG212transmission electron microscope (Philips, Amsterdam, Netherlands) was used to examine the ultrastructure of the stained sections.

### TUNEL assay

A TUNEL staining kit (Roche Molecular Biochemicals, Mannheim, Germany, Cat No#11,684,817,910) was used to identify apoptotic cells in situ. The labeling reaction, which contained terminal deoxynucleotidyl transferase, was carried out at 37 °C for 60 min in a humidity chamber after treatment with 10% goat serum in 0.1% Triton X-100 for 3 min. DAPI staining solution was used to counterstain the nuclei for 5 min at room temperature. The sections were cleaned with PBS and then examined with a laser scanning confocal microscope (Leica TCS SP8).

### ATP and MMP measurement

The luciferin-luciferase system was employed to quantify the ATP levels in these specimens according to the manufacturer’s instructions (Beyotime, Jiangsu, China, Cat.No#S0027). The relative ATP content of each specimen was determined by measuring the bioluminescent intensity with a microplate reader. A mitochondria isolation kit (Beyotime, Cat.No#C2006) was used to isolate mitochondria from each specimen in order to measure the MMP. Then, in accordance with the manufacturer’s instructions, the JC-1 probe was used to evaluate changes in MMP.

### Statistical analysis

Data are presented as mean ± standard deviation. One-way analysis of variance was used to determine statistical significance, and a post hoc least significant difference test was used to assess statistical differences between groups. SPSS 13.0 software was used to conduct the analyses (SPSS, Inc., Chicago, IL, USA). A statistically significant difference was defined as *P* < 0.05.

## Results

### Auditory impairment induced by D-gal

**Fig. 1:**
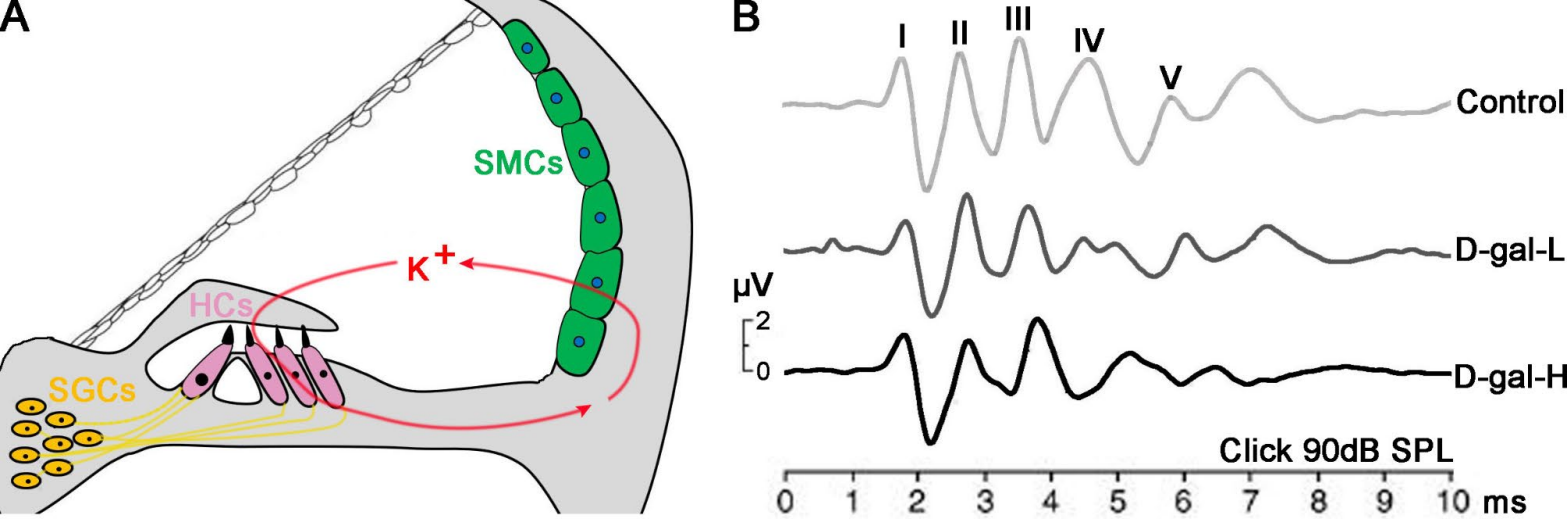
A schematic representation of the scala media and auditory function parameters of the mice in the control, D-gal-L, and D-gal-H groups. **(A)** Scheme showing that the Strial Marginal Cells (SMCs) in the lateral wall of the cochlea are critical for maintaining the K^+^ cycle and cochlear homeostasis. **(B)** Representative wave shapes of the ABR for the control, D-gal-L, and D-gal-H groups. The mean amplitudes of the ABR waves I–V were lower in the D-gal-L and D-gal-H groups than in the control group. The mean latency values of the ABR waves I–V were higher in the D-gal-L and D-gal-H groups than in the control group

The ABR test was used to assess the auditory function in control and D-gal-treated mice. Five vertex-positive peaks make up the mouse ABR waves. We discovered that the D-gal-induced ABR waves I–IV had a poorer amplitude and a longer latency than in the control mice (Fig. 1). Thus D-gal-induced aging does not lead to ABR threshold changes, but alters the amplitude and latency, which was particularly evident in the D-gal-H group.

### D-gal induces an increase in protein expression of NOX2, UCP2, and 8-OHdG in SV

**Fig. 2:**
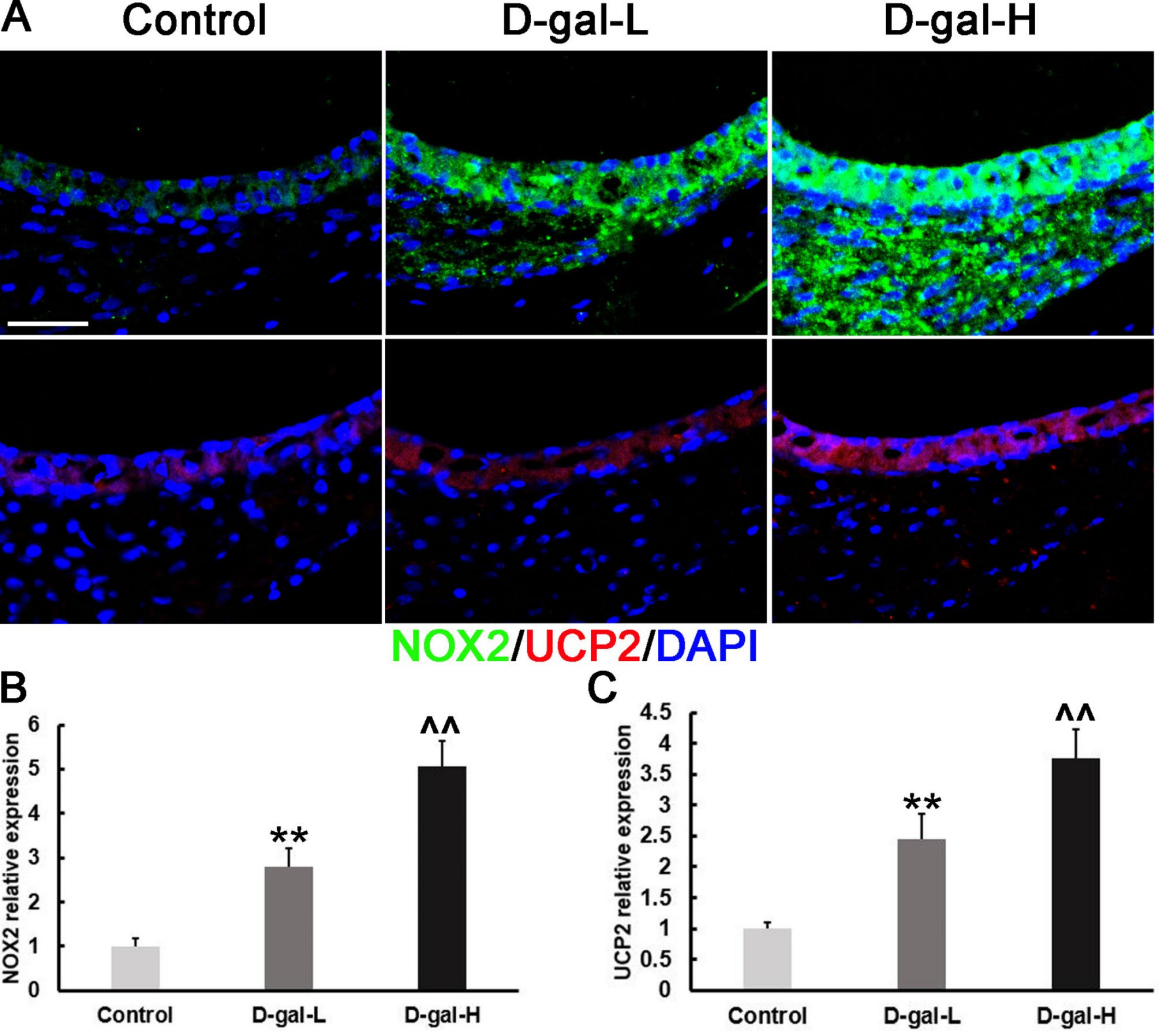
The NOX2 and UCP2 protein expression levels in the SV of the mice of different groups. **(A)** Representative images showing the expression and localization of NOX2 and UCP2 in the SV of mice in the control, D-gal-L, and D-gal-H groups. Scale bars = 25 μm. (Green: NOX2; red: UCP2; blue: DAPI). **(B)** NOX2 protein levels in the D-gal-L group were significantly higher than in the control group but lower compared to that of the D-gal-H group. **(C)** UCP2 protein levels in the SV of the mice of the D-gal-L group were significantly higher than those of the control group but lower compared to that of the D-gal-H group. Data are expressed as the mean ± SD of 6 mice per group. **, *P* < 0.01 vs. the control group; ^^, *P* < 0.01 vs. the D-gal-L group

**Fig. 3:**
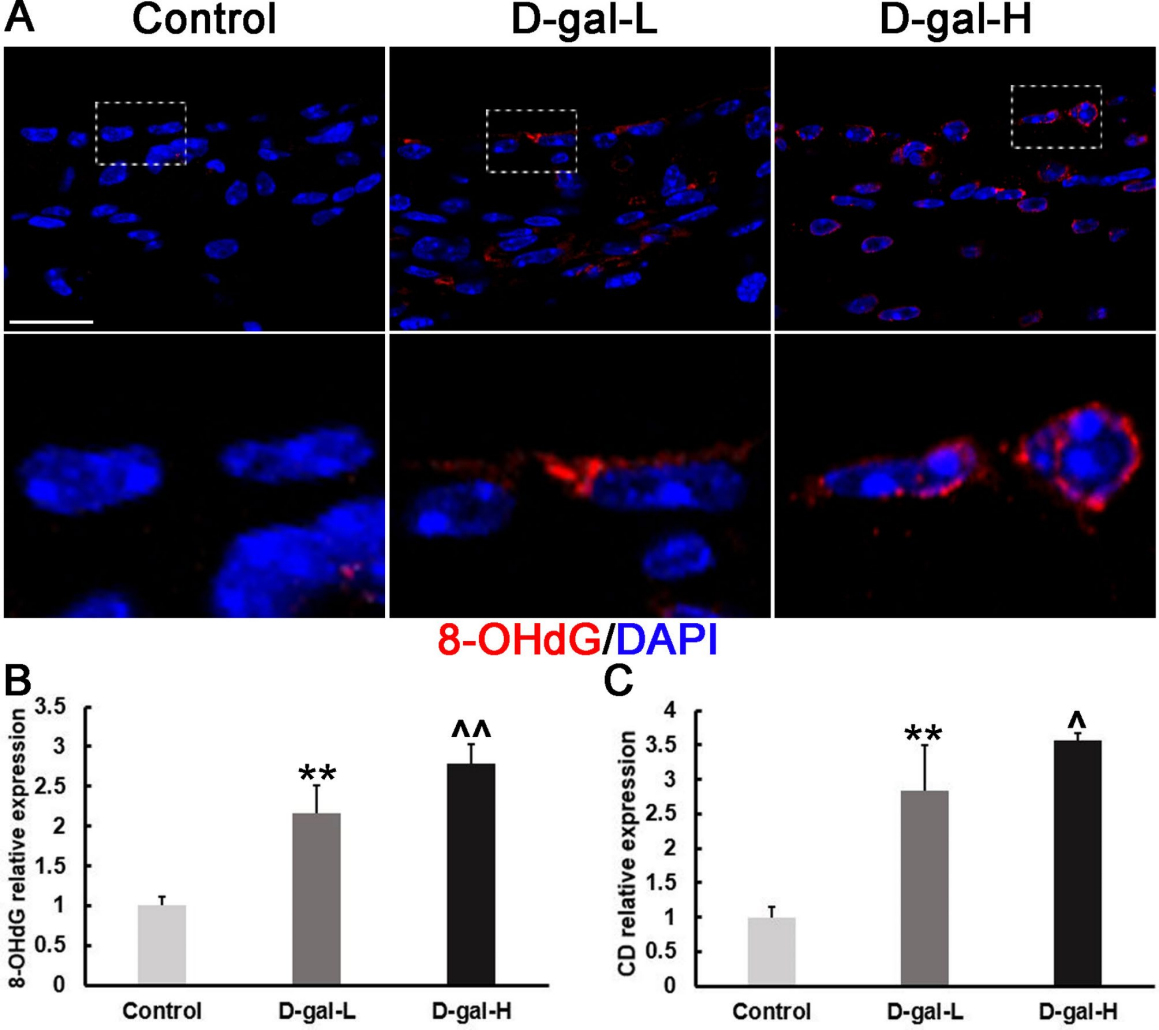
The 8-OHdG expression in the SV and quantification of the mtDNA CDs. **(A)** Representative images showing the expression and localization of 8-OHdG in the SV of mice in the control, D-gal-L, and D-gal-H groups. Scale bars = 25 μm. (Red: 8-OhdG; blue: DAPI). **(B)** The protein expression of 8-OHdG in the SV of the D-gal-L group was significantly higher than that of the control group mice but lower compared to that of the D-gal-H group. **(C)** The mtDNA CD level in the cochleae of mice in the D-gal-L group was significantly higher than that in the mice of the control group but lower compared to that of the D-gal-H group. Data are expressed as the mean ± SD of 6 mice per group. **, *P* < 0.01 vs. the control group; ^, *P* < 0.05 vs. the D-gal-L group; ^^, *P* < 0.01 vs. the D-gal-L group

Immunohistochemistry staining of cochlear SV showed that expression levels of NOX2, UCP2 and 8-OHdG proteins were significantly higher in the D-gal treatment groups than in the control group (Figs. 2 and 3). In addition, 8-OHdG was predominantly distributed in the cytoplasm of SV cells, revealed as red dotted fluorescence in Fig. 3A, and was more prevalent in the D-gal-H group.

### D-gal induces an increase in mtDNA CD level in SV

The mtDNA CD level in the cochlear SV was measured by qPCR. A dual-labeled fluorescent DNA probe was designed to specifically identify the fusion sequence, which was only present in mutant mtDNA that harbored CDs. As shown in Fig. 3C, the accumulation of mtDNA CDs was significantly higher in the D-gal-treated groups than in the control group (*P* < 0.01).

### D-gal induces a decrease in ATP level and MMP in cochlea

**Fig. 4:**
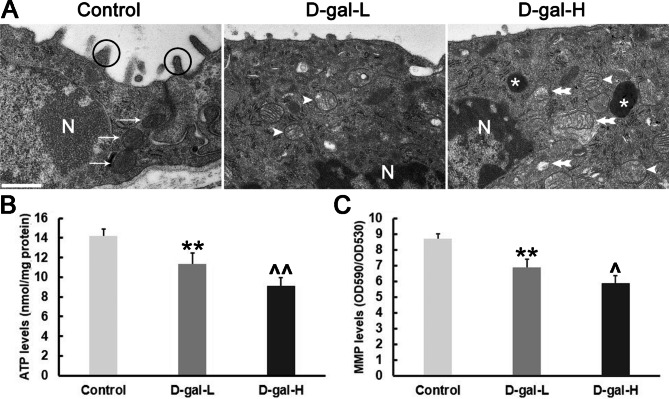
Alterations in the mitochondrial ultrastructure of the SV and quantification of the ATP level and MMP in the cochlea. **(A)** The mitochondria in the control group were normal (arrows), and those in the D-gal-L and D-gal-H groups had a swollen shape and reduced electron density in their matrix (arrowheads). Severely degenerated mitochondria are indicated with double arrows. In the control group, the microvilli in mitochondria of marginal cells were normal (circle); in the D-gal-L group, the microvilli were reduced or shortened and in the D-gal-H group, most microvilli were missing. Moreover, cytolysosomes containing mitochondrial remnants were found (asterisk) in the D-gal-H group. N, nucleus; n = 4 mice per group. Scale bar = 0.5 μm. **(B)** The ATP level in the cochleae of the mice in the D-gal-L group was significantly lower than that of the control group but significantly higher than that of the D-gal-L group. **(C)** The MMP in the cochleae of the mice in the D-gal-L group was significantly lower than that of the control group but significantly higher than that of the D-gal-L group. Data are expressed as the mean ± SD of 6 mice per group. **, *P* < 0.01 vs. the control group; ^, *P* < 0.05 vs. the D-gal-L group; ^^, *P* < 0.05 vs. the D-gal-L group

To further assess mitochondrial function, we measured ATP level and MMP using a colorimetric test kit. ATP levels were 14.17 ± 0.72, 11.33 ± 1.1 and 9.15 ± 0.8 9.15 ± 0.8 nmol/mg in control, D-gal-L and D-gal-H groups, respectively. ATP levels in the D-gal-L and D-gal-H groups were significantly lower than in control group (Fig. 4B). MMP production levels were 8.69 ± 0.31, 6.89 ± 0.5, and 5.87 ± 0.5 nmol/mg in control, D-gal-L, and D-gal-H groups, respectively. The MMP in the D-gal-L and D-gal-H groups were significantly lower than in control group (Fig. 4C).

### Alterations in the mitochondrial ultrastructure in the SV

To investigate changes in the ultrastructure of mitochondria in SV, we compared the mitochondrial morphology in the three groups (Fig. 4A). The TEM results revealed that the SV of the mice in the control group was rich in mitochondria, which had a normal shape and size; a with normal microvilli. In contrast, numerous mitochondria in the SV of mice in the D-gal-L and D-gal-H groups were swollen and had a reduced electron density in their matrix. In the D-gal-L group, the microvilli were reduced or shortened, and most microvilli were missing in the D-gal-H group. We also observed cytolysosomes containing mitochondrial remnants in the D-gal-H group.

### D-gal induces an increase in the c-Cas3 protein expression in the SV

**Fig. 5:**
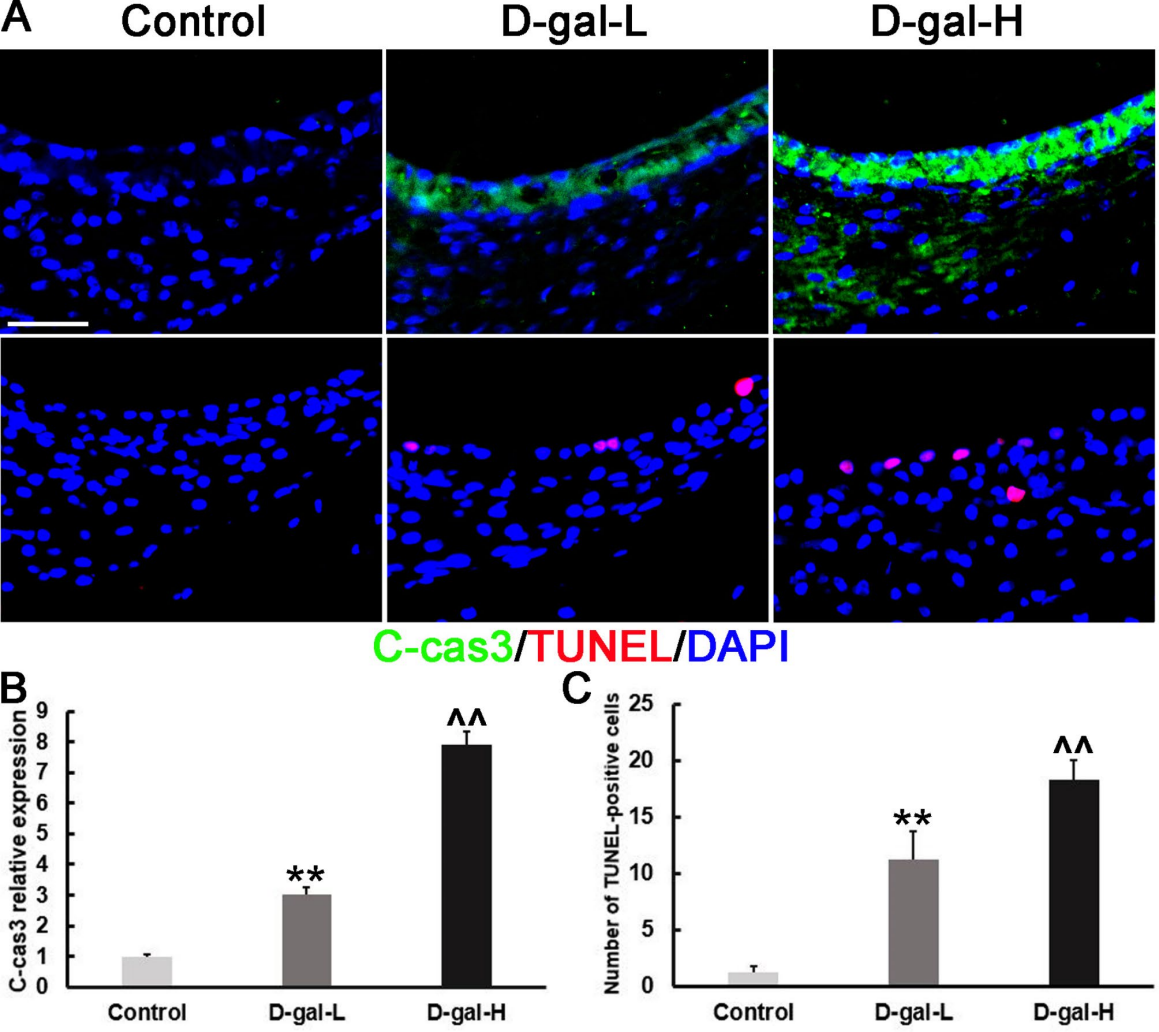
Changes in caspase-3-mediated apoptosis in the SV of mice. **(A)** Representative images showing the expression and localization of c-Cas3 protein and TUNEL-positive cells in the cochlear SV of mice in the control, D-gal-L, and D-gal-H groups. Scale bars = 25 μm. The c-Cas3 protein expression in the SV of the mice of the D-gal-L group was significantly higher than that of the control group but lower compared to that of the D-gal-H group. Compared with that of the D-gal-L group, a greater number of TUNEL-positive cells was found in the mice of the D-gal-H group; Almost no TUNEL-positive cells were found in the SV of mice in the control group. (Green: c-Cas3; red: TUNEL; blue: DAPI). **(B)** The expression of c-Cas3 protein in the SV of the mice in the D-gal-L group was significantly higher than those of the control group but lower compared to that of the D-gal-H group. **(C)** The number of TUNEL-positive cells in the SV of the mice of the D-gal-L group was significantly higher than that of the control group but lower compared to that of the D-gal-H group. Data are expressed as the mean ± SD of 6 mice per group. **, *P* < 0.01 vs. the control group; ^^, *P* < 0.01 vs. the D-gal-L group

The protein expression level of c-Cas3 in the cochlear SV was investigated by immunohistochemistry. As shown in Fig. 5A and B, its expression in the D-gal treated groups was significantly higher than in the control group.

### D-gal induces an increase in TUNEL-positive cells in the SV

Apoptotic activity was observed in the SV compartments of D-gal treated mice (Fig. 5A). The number of TUNEL-positive cells in the cochlear SV was significantly higher in the D-gal-treated groups than in the control group (Fig. 5C, P < 0.01).

## Discussion

Our main findings revealed that D-gal induced oxidative damage and apoptosis of SV mitochondria in mouse cochlea. According to the mitochondrial theory of aging, mitochondrial DNA is highly susceptible to oxidative damage during aging due to excess ROS production, decreased antioxidant levels, and a lack of protective histone proteins [27]. Du et al. found that the NOX2 system is a prominent generator of ROS in the cochlea of D-gal induced aging rats [11]. NOX2 is an enzyme complex that produces ROS by transferring electrons from NADPH to oxygen [28]. Also, it has been demonstrated that NOX2 is broadly distributed in various cells and organs, including the cochlea [29, 30]. Here, we discovered that NOX2 expression is increased in the cochlear SV of mice subjected to accelerated aging induced by D-gal (Fig. 2). Together, these data indicate that ROS generated by NOX2 may be, at least in part, responsible for the D-gal-induced mtDNA oxidative damage in the cochlear SV.

In addition to the NADPH oxidase system, mitochondria are another major source of ROS generation in vivo [27]. Mitochondrial ROS production can result in the activation of UCP2, which is localized to the inner mitochondrial membrane [31–33]. The level of cellular ROS is also indirectly reflected in UCP2 expression. Previous research had shown that rat models of D-gal-induced aging with overexpressed UCP2 in the cochlea and central auditory system [34]. In the current investigation, we discovered that UCP2 protein was significantly higher in the cochlea SV of mice treated with D-gal (Fig. 2). This finding suggests that mitochondrial ROS may be implicated in D-gal-induced mtDNA damage in the cochlea SV.

There is evidence that ROS overproduction in mitochondria can result in the progressive destruction of mtDNA [35]. One of the most common and best characterized age-associated mtDNA mutations is CD [36, 37]. CD, which is used as a molecular marker to evaluate age-induced DNA damage in the inner ear [10, 21]. Here, we found that D-gal significantly elevated CD levels of mtDNA in the cochlear SV (Fig. 3C). According to a recent study, the degree of hearing loss in presbycusis is significantly correlated with the CD level in the cochlea [38]. Therefore, the accumulation of the CD mutation is considered to play a crucial role in the development of ARHL. Moreover, the level of 8-OHdG, a biomarker of oxidative DNA damage, is also elevated and the high level of 8-OHdG in the cytoplasm, consistent with the view that D-gal administration leads to mtDNA oxidative damage in the cochlear SV (Fig. 3A, B). To further evaluate the mitochondrial damage induced by oxidative stress, we investigated the mitochondrial ultrastructural changes in the SV of cochleae. Swelling, deformation, vacuolization and lower electron density were found in numerous mitochondria in the marginal cells of the SV. In addition, the presence of cytolysosomes and the shortening and disappearance of microvilli further directly reflect the mitochondrial dysfunction (Fig. 4A).

Cell energy deficiency and mitochondrial malfunction may be driven by mtDNA mutations and ultrastructural damage. In the SV of aging mice induced by D-gal, we demonstrated that mitochondrial ATP production had decreased (Fig. 4B). It is well known that maintaining oxidative phosphorylation and ATP generation requires a normal MMP. The cumulative burden of ROS production can lead to MMP breakdown, which results in energy deficiency and mitochondrial dysfunction. The high accumulation of ROS leads to the destruction of MMP, which in turn leads to energy deficiency and mitochondrial malfunction. In the present study, both the D-gal-L and D-gal-H groups had a reduced MMP (Fig. 4C). The concomitant decline in MMP decreases the ATP levels in the cochlea, which is insufficient for the normal ion transportation in the inner ear, further inducing hearing loss [39].

Mitochondrial oxidative damage can initiate apoptosis. Apoptosis is initiated by two pathways: the intrinsic pathway, which is initiated by a change in mitochondrial membrane permeability, and the extrinsic pathway, which is activated by death receptors [40–42]. In the apoptosis intrinsic pathway, mitochondrial dysfunction can result in the permeabilization of the mitochondrial outer membrane, the release of cytochrome c into the cytosol, and activation of caspase-3 via proteolytic cleavage [43, 44]. To determine whether an increased level of c-Cas3 is a characteristic of the cochlear SV of mice subjected to D-gal-induced aging, the expression of c-Cas3 was evaluated through immunohistochemical analysis of frozen cochlear sections. Cleaved caspase-3 was elevated in the cochlear SV in both D-gal-L and D-gal-H treated mice. The expression of c-Cas3 was higher in the latter group than in the former (Fig. 5). DNA fragmentation is associated with apoptosis. We found that TUNEL-positive cells in the cochlear SV were increased significantly in the D-gal-treated groups. Although we observed a higher number of apoptotic cells in the SV of the cochleae (Fig. 5), this damage in the peripheral auditory system might be insufficient to explain the hearing impairment [6].

Taken together, these results indicate that mitochondrial oxidative damage and the subsequent apoptosis may be responsible for cochlear impairment. D-gal induces mitochondrial dysfunction, which in turn, increases ROS production. A considerable amount of ROS can trigger progressive oxidative damage, promote the oxidation of mtDNA and associated proteins [45], which can result in apoptosis and impairment of the normal function of the cochleae. In recent years, some researchers have found that autophagy can attenuate ROS accumulation and alleviate age- and cisplatin-induced oxidative stress in the auditory systems [4]. Thus, in the process of degeneration of the auditory system, hair cells can protect themselves from a certain level of damage by activating autophagy. Our results provide evidence that supports the existence of a NOX–ROS–DNA damage linkage and indicate that this process leads to apoptosis of marginal cells, further influencing cochlear microcirculation and inducing hearing loss. Also, the current study confirms that oxidative damage to cochlear SV marginal cells is one of the main mechanisms in the D-gal-induced aging model, providing a theoretical basis for subsequent antioxidant therapy.

## Data Availability

The datasets used and/or analyzed during the current study are available from the corresponding author on reasonable request.

## Abbreviations

ARHL: Age-related hearing loss
SV: Stria vascularis
D-gal: D-galactose
mtDNA: Mitochondrial DNA
CD: Common deletion
NOX2: Nitrous oxide
UCP2: Uncoupling protein 2
ATP: Adenosine triphosphate
MMP: Mitochondrial membrane potential
8-OHdG: 8-hydroxy-2-deoxyguanosine
ABR: Auditory brainstem response
TM: Tympanic membrane
AOE: Acute otitis externa
ROS: Reactive oxygen species
TUNEL: Terminal deoxynucleotidyl transferase-mediated dUTP-biotin nick end-labeling
